# Cytoplasmic BRMS1 expression in malignant melanoma is associated with increased disease-free survival

**DOI:** 10.1186/1471-2407-12-73

**Published:** 2012-02-22

**Authors:** Ana Slipicevic, Ruth Holm, Elisabeth Emilsen, Anne Katrine Ree Rosnes, Danny R Welch, Gunhild M Mælandsmo, Vivi Ann Flørenes

**Affiliations:** 1 Department of Pathology, Oslo University Hospital, The Norwegian Radium Hospital, Oslo, Norway; 2 Department of Cancer Biology, The Kansas University Medical Center, Kansas City, USA; 3 Department of Tumor Biology, Institute for Cancer Research, Oslo University Hospital The Norwegian Radium Hospital, Oslo, Norway; 4 Department of Pharmacy, Faculty of Health Sciences, University of Tromsø, Tromsø, Norway

## Abstract

**Background/aims:**

Breast cancer metastasis suppressor 1 (BRMS1) blocks metastasis in melanoma xenografts; however, its usefulness as a biomarker in human melanomas has not been widely studied. The goal was to measure BRMS1 expression in benign nevi, primary and metastatic melanomas and evaluate its impact on disease progression and prognosis.

**Methods:**

Paraffin-embedded tissue from 155 primary melanomas, 69 metastases and 15 nevi was examined for BRMS1 expression using immunohistochemistry. siRNA mediated BRMS1 down-regulation was used to study impact on invasion and migration in melanoma cell lines.

**Results:**

A significantly higher percentage of nevi (87%), compared to primary melanomas (20%) and metastases (48%), expressed BRMS1 in the nucelus (p < 0.0001). Strong nuclear staining intensity was observed in 67% of nevi, and in 9% and 24% of the primary and metastatic melanomas, respectively (p < 0.0001). Comparable cytoplasmic expression was observed (nevi; 87%, primaries; 86%, metastases; 72%). However, a decline in cytoplasmic staining intensity was observed in metastases compared to nevi and primary tumors (26%, 47%, and 58%, respectively, p < 0.0001). Score index (percentage immunopositive celles multiplied with staining intensity) revealed that high cytoplasmic score index (≥ 4) was associated with thinner tumors (p = 0.04), lack of ulceration (p = 0.02) and increased disease-free survival (p = 0.036). When intensity and percentage BRMS1 positive cells were analyzed separately, intensity remained associated with tumor thickness (p = 0.024) and ulceration (p = 0.004) but was inversely associated with expression of proliferation markers (cyclin D3 (p = 0.008), cyclin A (p = 0.007), and p21^Waf1/Cip1 ^(p = 0.009)). Cytoplasmic score index was inversely associated with nuclear p-Akt (p = 0.013) and positively associated with cytoplasmic p-ERK1/2 expression (p = 0.033). Nuclear BRMS1 expression in ≥ 10% of primary melanoma cells was associated with thicker tumors (p = 0.016) and decreased relapse-free period (p = 0.043). Nuclear BRMS1 was associated with expression of fatty acid binding protein 7 (FABP7; p = 0.011), a marker of invasion in melanomas. In line with this, repression of BRMS1 expression reduced the ability of melanoma cells to migrate and invade *in vitro*.

**Conclusion:**

Our data suggest that BRMS1 is localized in cytoplasm and nucleus of melanocytic cells and that cellular localization determines its *in vivo *effect. We hypothesize that cytoplasmic BRMS1 restricts melanoma progression while nuclear BRMS1 possibly promotes melanoma cell invasion.

Please see related article: http://www.biomedcentral.com/1741-7015/10/19

## Introduction

Although melanomas accounts for only 4% of all dermatological cancers, they are responsible for approximately 80% of skin cancer-related deaths. In early stages, melanomas can be treated surgically and 5-year survival rate exceed 80%. However, less than 15% of patients having metastatic disease (stage IV) can expect to survive 5 years as there are few or no therapeutic options. The molecular mechanisms responsible for melanoma development and progression are not completely understood. Thus, novel diagnostic and prognostic biomarkers as well as improved treatment strategies are urgently needed [1].

Breast cancer metastasis suppressor 1 (BRMS1) was originally identified following differential expression comparisons of chromosome 11 microcell hybrids in a human breast carcinoma cell line and was further mapped to chromosome fragment 11q13, a region frequently altered in melanomas [2]. Re-expression of BRMS1 in human breast, non-small cell lung (NSCL) and ovarian carcinomas and in melanoma cell lines resulted in marked reduction of metastasis without blocking orthotopic tumor growth [3].

Although BRMS1 has been described as a predominantly nuclear protein, it has recently become clear that it is also localized in the cytoplasm [4,5]. As part of the Sin3:histone deacetylase transcription complex [6], BRMS1 affects metastasis by directly or indirectly repressing expression of pro-metastatic genes and enhancing the expression of other anti-metastatic genes [7]. Furthermore, BRMS1 differentially regulates cancer cell responses to growth factor signaling. Thus, BRMS1 reduces downstream PI3-kinase/Akt signaling by altering phosphoinositide pools and/or by inhibiting epidermal growth factor receptor expression [8]. BRMS1 also represses NF-κB transcriptional activity, urokinase-type plasminogen activator and osteopontin [9] expression, as well as upregulates expression of anti-metastatic microRNA [10]. In breast, ovarian and NSCL carcinomas, BRMS1 suppresses metastasis by inhibiting growth initiation at secondary sites (i.e., colonization) without preventing primary tumor growth [11,12]. In NSCLC, BRMS1 expression decreased migration and invasion [12]. Similarly, melanoma cells re-expressing BRMS1 were less invasive and had restored intercellular communication [13].

In a subgroup of patients suffering from breast cancer, loss of tumor expression of BRMS1 protein was associated with reduced disease-free survival [14]. Furthermore, a shift from nuclear to cytoplasmic BRMS1 expression was found associated with highly proliferative estrogen receptor negative breast cancers [4]. In NSCLC, expression of BRMS1 protein was associated with increased patient survival [12]. Recently, Li *et al. *[15] observed decreased BRMS1 protein in metastatic melanomas compared to benign nevi and primary tumors as well as an association with tumor stages and worse prognosis. In contrast to these findings, Kelly *et al. *[16] found no correlation between BRMS1 mRNA level and breast cancer metastasis to regional lymph nodes, while Lombardi *et al. *[17] showed that high BRMS1 mRNA expression correlates with poor prognosis for patients with breast cancer. Although the mRNA findings are interesting, we have previously reported that mRNA and protein levels for BRMS1 do not always correlate [18]; therefore, interpretation should be done with caution. Nonetheless, although recent literature suggest that BRMS1 may play roles during tumor progression other than the metastasis suppressor function originally suggested, this study was undertaken to investigate the importance of BRMS1 expression and subcellular localization on melanoma progression.

## Materials and methods

### Clinical melanoma specimens

Formalin-fixed, paraffin-embedded tissue from 155 primary melanomas and 69 metastases, as well as 15 benign nevi, was examined for expression of BRMS1 protein. Of the primaries, 93 were classified as superficial spreading (SSM) and 62 as nodular melanoma (NM). Clinical follow-up was available for all patients. The study was approved by the Regional Committee for medical Research Ethics in Norway.

### Immunohistochemical analysis

Three-μm sections made from formalin-fixed paraffin embedded tissues were immunostained using the Dako EnVision™+ system (K8012, Dako Cooperation, CA, USA). Deparaffinization, rehydration and target retrieval were performed in one operation in a Dako PT-link and EnVision™ Flex target retrieval solution with high pH. To block endogenous peroxidase the sections were treated with Dako blocking reagent for 5 minutes. Sections were incubated with monoclonal BRMS1 antibody (clone 3a1.21) (supplied by Dr. Welch) diluted 1:500 (2 μg IgG_1_/mL) [19] for 30 minutes. Thereafter, the sections were incubated with Dako EnVision™ FLEX+ mouse linker for 15 minutes followed by incubation with Dako EnVision™ FLEX/HRP for an additional 30 minutes. For visualization of staining, the sections were treated with AEC+ High sensitivity Substrate Chromogen Ready-to-use (K3469, Dako) (paraffin-embedded tissue) or 3'3-diaminobenzidine tetra-hydrochloride (DAB) (cell lines), counterstained with haematoxylin and mounted from water in Dako Aqueous Mounting Medium Ready-to-use. Sections from normal skin with known expression of BRMS1 was used as positive control, whereas negative controls included substitution of monoclonal antibody with mouse myeloma protein of the same subclass and concentration as the monoclonal antibody. Four semi-quantitative classes were used to describe staining intensity (absent, 0; weak, 1; moderate, 2; strong, 3) and percentage of positive tumor cell: absent, 0; < 10%, 1; 10-50%, 2; > 50%, 3). By multiplying intensity score with extent score, a score index was calculated ranging from 0 to 9. Staining in cytoplasm and nucleus were evaluated separately. BRMS1 expression in more than 10% of the tumor cells was considered as high percentage, while moderate and strong staining intensity were considered as strong. Similarly, a score index of ≥ 4 was considered as high. Immunohistochemical staining of cyclin D1, cyclin D3, cyclin A, p21^Waf1/Cip1 ^p27^Kip1^, p-ERK1/2, p-Akt, and FABP7 has been performed previously [20-26].

### Cell cultures and small interfering RNA (siRNA) transfection

The WM239 cell line was kindly provided by Dr. Meenhard Herlyn, (Wistar Institute, Philadelphia, PA) whereas the FEMX-1 cell line was established from a lymph node metastasis obtained from a melanoma patient treated at the Norwegian Radium Hospital, Oslo University Hospital [27]. 1 × 10^6 ^cells were plated 24 hrs prior to transfection in T75 culture flasks containing RPMI 1640 medium (Lonza, Verviers, Belgium) supplemented with 5% foetal bovine serum (FBS) (PAA Laboratories, GMbH, Austria) and 2 mM L-glutamine (GibcoBRL, Paisley, UK). The cells were transfected with 25 nM siRNA targeting BRMS1 (OligoID: HSS177871) or stealth RNAi siRNA negative control in 37.5 uL Lipofectamine 2000 according to the manufactures instructions (Invitrogen, Carlsbad, CA). The siRNA/lipofectamine 2000 mixture was added in a total of 10 mL OptiMEM to each flask (all reagents and siRNA were from Invitrogen. Six hrs after transfection, medium was replaced with10 mL complete medium and 1 mL [^3^H] Thymidine (ARC St. Louis, MO) was added to the cell cultures for migration and invasion assays. Twenty-four hrs thereafter, the cells were detached by trypsinization, counted and seeded in invasion chambers.

### Quantitative real time RT-PCR

For mRNA expression analyses, the cells were detached 48 hrs after treatment with siRNA. Total RNA was extracted using Trizol reagent (Invitrogen,) according to manufacturer's description and reverse transcribed with the high capacity cDNA reverse transcription kit (Applied Biosystems, Foster City, CA) using random primers. The real time RT-PCR analyses were performed as previously described [26] with TaqMan Gene Expression Assays (Hs00363036_m1 BRMS1, Hs99999908_m1 GUSB, Applied Biosystems). The relative BRMS1 mRNA expression levels were normalized against housekeeping gene beta-glucuronidase (GUSB). Each sample was run in triplicate. The mean from three independent experiments was calculated.

### Migration and invasion assays

Migration and invasive properties were evaluated in a 24 well transwell chamber assay, 8 μM pore size (Costar, Cambridge, MA). For analyzing invasiveness, the filters were coated with 25 μg/filter Matrigel (BD Biosciences, Bedford, MA) whereas migration was analyzed using uncoated filters. 5 × 10^4 ^[^3^H] Thymidine-labeled cells in 100 μL RPMI 1640 medium were applied in the upper compartments, in triplicate wells. The lower compartments were supplied with 600 μL RPMI 1640 medium and 5% FBS as chemo attractant. After 48 hrs, the cells on the upper and lower parts of the filters were removed separately using cotton-tipped swabs, and incorporated [^3^H] Thymidine was counted in a liquid scintillation analyzer (Packard Instrument Company, Chicago, IL). Migration/invasion was assessed as the ratio of counts obtained from the lower compartments compared to total counts of both compartments.

### Statistical analysis

A comparison between variables was performed using the χ^2 ^test or the Fisher exact test. The relationship between BRMS1 expression and mean tumor thickness was evaluated using the Mann-Whitney 2-sample test. Kaplan- Meier survival estimates and log-rank tests were used to evaluate the survival data. Two-tailed paired Student's t-test was used to evaluate the *in vitro *results. A p-value of less than 0.05 was considered statistically significant.

## Results

**Figure 1:**
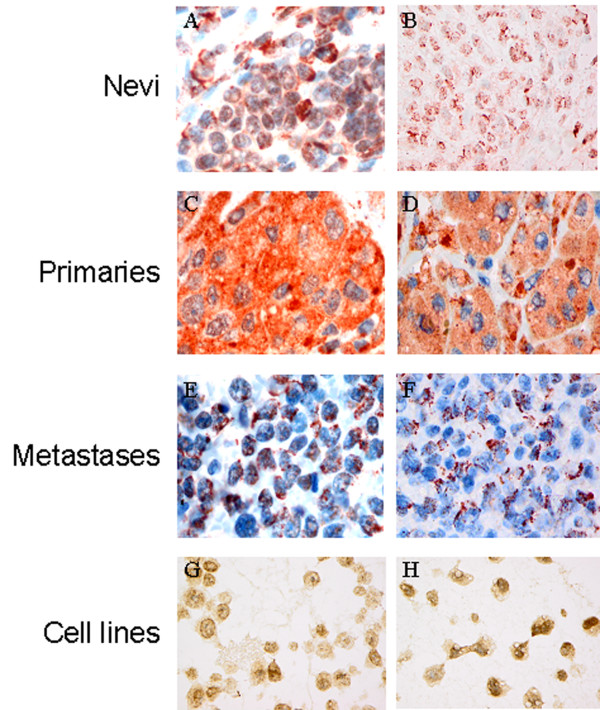
**Immunohistochemical staining of BRMS1 in benign nevi showing cytoplasmic and nuclear (A,) and nuclear only expression (B), primary melanomas with cytoplasmic expression (C, D) and metastatic melanomas demonstrating cytoplasmic and nuclear (E) and cytoplasmic only immunoreactivity (F)**. In the two melanoma cell lines (G, H) BRMS1 is localized mainly in the nucleus, although some cytoplasmic expression is also present.

Protein expression of BRMS1 was analyzed by immunohistochemistry in a panel of paraffin-embedded benign nevi and primary and metastatic melanoma tissues as well as in two melanoma cell lines. Heterogeneous cytoplasmic and/or nuclear expression was observed. However, BRMS1 immunoreactivity was not found to accumulate in specific parts of the tumor biopsies. The results are summarized in Table 1 and illustrated in Figure 1. A significantly higher percentage of benign nevi (87%) as compared to primary (20%) and metastatic melanoma (48%), expressed BRMS1 in the nucleus (p < 0.0001). Cytoplasmic expression was found in 87% of benign nevi and in 86% and 72% of primaries and metastases, respectively. When staining intensity was evaluated separately, strong nuclear intensity was observed in 67% of nevi but in only 9% of primary and 24% of metastatic tumors (p < 0.0001). Whereas 47% of the nevi and 58% of the primaries showed strong cytoplasmic staining intensity, a significant decline was observed in the metastases (26%; p < 0.0001). When score index (percentage of immunoreactive cells multiplied with staining intensity) was used as readout, high nuclear score index (≥ 4) was observed in 53% of nevi as compared to only 5% of the primary tumors. Interestingly, the opposite was observed for cytoplasmic staining; high cytoplasmic score index was seen in 50% of the primaries but in only 7% of the nevi. Nuclear and cytoplasmic score indexes were comparable in the metastases (22% and 19%, respectively) (Table 1). In melanoma cell lines, BRMS1 was localized mainly in the nucleus, although some cytoplasmic expression was also seen (Figure 1G, H).

**Table 1:** Expression of BRMS1 according to number of positive cases, intensity and subcellular localization

	^1^No. positive (%)			Intensity (%)		Score index (%)	
	
				Nucleus	Cytoplasm	Nucleus	Cytoplasm
	
	Total	Nucleus	Cytoplasm	**Strong**^**2**^	Strong	**High**^**3**^	High
Nevi	15 (100)	13 (87)	13 (87)	10 (67)	7 (47)	8 (53)	1 (7)

Primary Melanomas	136 (88)	32 (20)	134 (86)	14 (9)	90 (58)	7 (5)	77 (50)

SSM^4^	83 (89)	17 (20)	82 (88)	7 (8)	61 (66)	2 (2)	54 (58)

NM^5^	53 (85)	15 (24)	52 (84)	7 (11)	29 (45)	5 (8)	23 (37)

Metastases	62 (90)	33 (48)	50 (72)	17 (24)	18 (26)	15 (22)	13 (19)

^1^Includes all positive cases

^2^Strong staining intensity includes moderate and strong intensity

^3^High score index defined as ≥ 4

^4^SSM; Superficial spreading melanoma

^5^NM; Nodular melanoma

### Expression of BRMS1 in relation to clinical parameters

**Figure 2:**
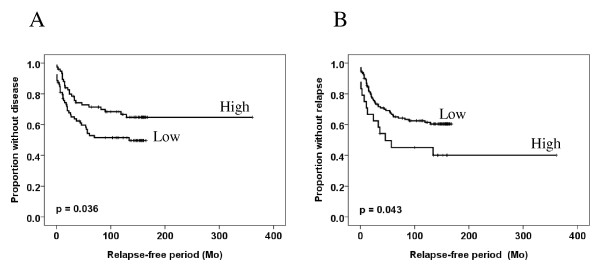
**Kaplan-Meier curve demonstrating relationship between BRMS1 cytoplasmic score index (A) and between percentage of cells expressing nuclear BRMS1 (B) and disease-free survival**.

BRMS1 expression levels in superficial spreading and nodular melanomas revealed no obvious differences with regard to score index, number of immunoreactive cells, nor staining intensity (Table 1). Moreover, no differences were observed when comparing radial (tumor thickness < 1 mm) and vertical (tumor thickness ≥ 1 mm) growth phase tumors. (data not shown). High cytoplasmic BRMS1 score index was significantly associated with thinner tumors (p = 0.040), lack of ulceration (p = 0.020), and increased disease-free survival (p = 0.036; Table 2 and Figure 2A). However, only staining intensity remained associated with tumor thickness (p = 0.024) and ulceration (p = 0.004) when intensity and percentage BRMS1 immunoreactive cells were analyzed separately (Table 3). Tumors with high percentage of cells expressing nuclear BRMS1 were thicker (p = 0.016) (Table 4) and had shorter relapse-free survival (p = 0.043) (Figure 2B). Neither nuclear score index nor staining intensity were associated with any of the examined clinical parameters (data not shown). Neither cytoplasmic nor nuclear BRMS1 expression had impact on overall survival (data not shown).

**Table 2:** Relationship between cytoplasmic BRMS1 score index, ulceration, tumor thickness, cyclin D3 and activation of ERK1/2 and Akt

		Cytoplasmic BRMS1 score index		
**Clinical parameter**	**Expression**	**Low**	**High**^a^	**P**

Mean tumor depth (mm)		3.60 (3.29)^b^	2.42 (2.46)	0.040

	No	41	53	
Ulceration	Yes	30	16	0.020

**Marker**				

Cyclin D3	Low	42	57	
	High	32	12	0.001

	Low	43	29	
Cytoplasmic p-ERK	High	32	46	0.033

	Low	46	59	0.013
Nuclear p-Akt	High	26	12	

^a^High score index defined as ≥ 4

Low protein expression was defined as immunoreactivity in less than 5% (cyclin D3) [21]

in < 50% of the tumor cells (p-Akt) [25], or total lack of expression (p-ERK) [23]

^b^SD

**Table 3:** Relationship between intensity of BRMS1 expression, ulceration, tumor thickness and markers of proliferation

		Intensity of cytoplasmic BRMS1 staining		
**Clinical parameter**	**Expression**	**Weak**	**Strong**^a^	**P**

Mean tumor depth (mm)		3.26 (3.11)^b^	1.92 (1.89)	0.024

Ulceration	No	71	23	
	Yes	44	2	0.004

**Marker**				

	Low	53	19	
Cyclin A	High	65	6	0.007

	Low	70	20	
Ki67	High	48	5	0.068

Cyclin D3	Low	76	23	
	High	42	2	0.008

	Low	32	17	
p21	High	31	3	0.009

^a^Low protein expression was defined as immunoreactivity in less than 5% of the tumor cells

(cyclin A, Ki67, cyclin D3, p21)[21,22,24]

^b^SD

**Table 4:** Relationship between nuclear BRMS1 expression, tumor thickness, and expression of FABP7

		Expression of nuclear BRMS1		
**Clinical parameter**	**Expression**	**Low**^**a**^	**High**	**P**

Mean tumor depth (mm)		2.72 (2.69)^c^	4.70 (3.85)	0.016

**Marker**				

	Low^b^	52	1	
FABP7	High	74	15	0.011

^a ^Low when < 10% of the tumor cells express BRMS1

^b^Low when < 5% of the tumor cells express FABP7 [26]

^c^SD

### Relationship between cytoplasmic BRMS1 expression and markers of proliferation and signal transduction

Since this panel of melanoma tissues has been previously analyzed for factors involved in cell proliferation [20-22,24], it was of interest to examine the relationship between BRMS1 expression and the levels of those factors. A significant inverse correlation between cytoplasmic BRMS1 score index and cyclin D3 expression was observed (p = 0.001) (Table 2). Interestingly, however, when analyzing percentage cytoplasmic BRMS1 positivity and staining intensity separately, a strong inverse association was found between staining intensity and cyclin D3 (p = 0.008), cyclin A (p = 0.007), and p21^Waf1/Cip1 ^(p = 0.009) (Table 3). Furthermore, although not significant, we found a negative association between BRMS1 staining intensity and Ki67 expression (p = 0.068) (Table 3). No correlation was seen between cytoplasmic BRMS1 staining intensity and cyclin D1 or p27^Kip1 ^expression (data not shown). Nuclear BRMS1 expression was not associated with markers of proliferation (data not shown).

It was demonstrated previously that expression of BRMS1 is correlated with activation of the PI3-kinase/Akt and NF-κB signaling pathways [8,9,28]. Here we present results showing that cytoplamic score index is inversely correlated to nuclear p-Akt expression (p = 0.013), but positively associated with expression of activated cytoplasmic ERK1/2 (p = 0.033) (Table 2).

### Nuclear BRMS1 expression is associated with invasive properties

We have recently demonstrated an association between protein expression of fatty acid binding protein 7 (FABP7) and proliferation and invasion of melanoma cells [26]. When examining the melanoma panel for a possible association between BRMS1 and FABP7, a significant correlation between nuclear BRMS1 expression (percentage of immunoreactive cells) and the level of FABP7 (p = 0.011) (Table 4) was observed.

**Figure 3:**
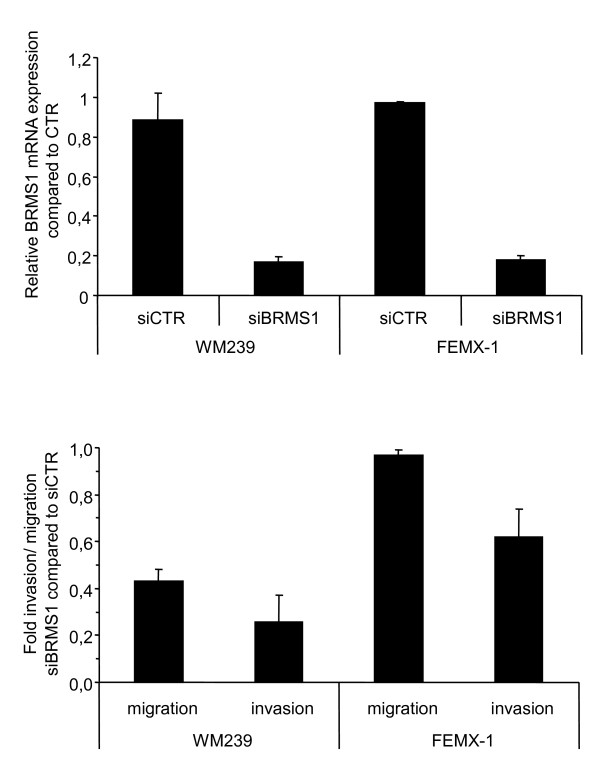
**Effect of BRMS1 on migration and invasion of melanoma cells**. BRMS1 expression was transiently down-regulated using siRNA in the metastatic melanoma cell lines WM239 and FEMX-1 (A) and analyzed for migration and invasion ability in a transwell chamber assay (B). Bars represent mean ratio of cells ± SE of cells in the lower compartment compared to total number of cells in both compartments from at least three independent biological experiments. WM239: invasion; p = 0.047, migration; p = 0.013, FEMX-1; invasion; p = 0.016, migration; p = 0.43.

To examine in more detail whether BRMS1 affects migration and invasion of melanoma cells we transiently down-regulated BRMS1 expression using siRNA in two metastatic melanoma cell lines (WM239, FEMX-1) (Figure 3A) and performed a transwell chamber invasion/migration assay. As demonstrated in Figure 3B, repression of BRMS1 significantly reduced invasion in both cell lines (WM239; p = 0.047, FEMX-1; p = 0.016) whereas migration was only repressed in the WM239 cells (WM239; p = 0.013, FEMX-1; p = 0.43).

## Discussion

Immunohistochemistry was applied to examine the level of BRMS1 protein in a panel of benign nevi and primary and metastatic melanomas in order to evaluate the impact of altered expression on clinical outcome. In accordance with previous studies demonstrating higher mRNA and/or nuclear BRMS1 protein expression in normal or benign tissues as compared to malignant tumors [4,12,29], BRMS1 was highly expressed in the nucleus of benign nevi whereas only a minor fraction of primary melanomas or metastases showed a similar expression profile. Our findings also partially agree with the study by Li *et al. *[15] who observed a decline in nuclear BRMS1 expression from dysplastic nevi to primary melanomas. A possible explanation for the more dramatic decline in our study may be that, in the previous study, malignant lesions were compared to dysplastic nevi while benign nevi of compound and intradermal subtypes where utilized in our investigations. Thus, it may be speculated that loss of nuclear BRMS1 expression is an early event, distinguishing common and dysplastic nevi.

Although BRMS1 has been recognized as a mainly nuclear protein, Rivera *et al. *[5] recently showed that BRMS1 contains both nuclear import and export signals, and implied that nuclear-cytoplasmic shuttling may represent a novel mechanism for altering the activity or function of BRMS1. The data in this study and a recent immunocytochemistry report in breast cancer [4] are consistent with that hypothesis. During melanocyte transformation and progression, BRMS1 appears to relocalize from the nucleus to the cytoplasm. Additionally, cytoplasmic staining intensity appeared to have a more pronounced impact than number of immunoreactive cells, suggesting that the level of BRMS1 protein has to reach a threshold level to have an effect on cellular behavior. Although there was no obvious difference in the percentage of tumor cells expressing BRMS1 in the cytoplasm, there was a clear decline in staining intensity in metastases as compared to nevi and primary tumors. Our findings are, however, in contrast to the study by Li *et al*., [15] who observed predominantly nuclear BRMS1 expression in benign and malignant melanocytic cells. One explanation for this discrepancy may be the use of different antigen retrieval and detection methods.

Frolova *et al. *[4] reported that cytoplasmic BRMS1 was associated with increased proliferation in estrogen receptor-negative breast cancers. In contrast, the melanoma samples used here show that cytoplasmic expression of BRMS1 is inversely associated with markers of proliferation, cyclin D3, cyclin A, Ki67, p21^Waf1/Cip1^. When coupled with our previously demonstrated associations between cell cycle regulators, numbers of mitosis and disease progression [21,22,24], the data presented further strengthen the hypothesis that cytoplasmic BRMS1 is associated with a less aggressive melanoma phenotype (i.e., thinner tumors, less ulcerated, longer survival).

ERK1/2, when sequestered in the cytoplasm, has been suggested to prevent transcription of pro-survival and proliferative proteins as well as enhance the activity of pro-apoptotic cytoplasmic proteins [30]. Furthermore, whereas Gayer *et al. *[31] recently suggested that excluding ERK1/2 from the nucleus inhibits proliferation, Jovanovic *et al*.[32] observed that the presence of activated ERK1/2 in the cytoplasm of melanomas was associated with better prognosis. In line with this, we demonstrated a strong positive association between BRMS1 and activated ERK1/2 when both were localized in the cytoplasm. Moreover, we also observed a strong inverse association between cytoplasmic BRMS1 expression and accumulation of activated p-Akt in the nucleus. Prior studies observed a negative association between BRMS1 and activation of PI3-kinase/Akt signaling [28,33], which is consistent with the well documented evidence that PI3-kinase/Akt signaling affects numerous steps of the metastatic cascade, including proliferation, apoptosis, migration and invasion [34]. In thyroid cancer, nuclear localization of activated Akt was associated with tumor invasion and metastasis [35]. Together these results suggest that cytoplasmic BRMS1 may at least partly negatively regulate melanoma progression and metastasis through sequestering of activated ERK1/2 in the cytoplasm and by preventing accumulation of nuclear active Akt.

In contrast to the results obtained in another melanoma cohort [15], as well as in breast [14] and NSCL cancers [12] our study showed that high nuclear expression of BRMS1 was associated with more aggressive tumors and shorter disease-free survival. The higher nuclear expression of BRMS1 found in benign nevi than in primary melanomas or metastases seems contradictory to the association to prolonged disease-free survival for patients having low nuclear tumor expression of BRMS1. As yet, we are unable to explain this phenomenon, but benign nevi are commonly terminal lesions as opposed to melanomas. Thus, the molecular events regulating these processes might differ. In this regard it has been shown that mutated B-Raf plays a role in inducing senescence in melanocytes, whereas in melanomas it contributes to oncogenesis [36]. Furthermore, in a recent study we showed that expression of fatty acid binding protein 7 (FABP7) is higher in benign nevi than in melanomas, still FABP7 was suggested to contribute to disease progression, most likely by increasing tumor cell invasion [26]. Interestingly, we observed a strong positive association between nuclear BRMS1 and FABP7 expression, suggesting that nuclear BRMS1 may, in fact, increase the invasive potential. In support of this, we showed that down-regulation of BRMS1 in two metastatic melanoma cell lines, expressing predominantly nuclear BRMS1, reduced the invasive ability.

Although it has not yet been verified at the protein level, the mRNA for various splice variants that are differently expressed in metastatic and non-metastatic breast cancer cell lines have been identified [18,37]. Thus, it may be speculated that benign nevi and primary and metastatic melanomas express different BRMS1 variants with different biological functions.

## Conclusion

The BRMS1 metastasis suppressor is localized in both the cytoplasm and nucleus of melanocytic tumors. Cellular localization corresponds to different effects, cytoplasmic BRMS1 appears to restrict tumor progression by negatively affecting cell proliferation, sequestering p-ERK1/2 in the cytoplasm and by preventing accumulation of nuclear p-Akt while nuclear BRMS1 may promote melanoma invasion, perhaps by its association with FABP7. Clearly, while BRMS1 may have shared roles in some tumor types, it is apparent that those roles can vary by the cell type from which a tumor arises and also by the tumor microenvironment.

## Competing interests

The authors declare that they have no competing interests.

## Authors' contributions

AS and EE optimized and performed the immunohistochemical analyses. RH evaluated the immunohistochemical staining. EE and AKRR performed the siRNA transfections and the invasion and migration assays. VAF conceived the study and drafted the manuscript. GMM participated in the design and coordination of the study whereas DW provided the antibody. All authors have read and approved the final manuscript.

## Pre-publication history

The pre-publication history for this paper can be accessed here:

http://www.biomedcentral.com/1471-2407/12/73/prepub
